# Different waves and directions of Neolithic migrations in the Armenian Highland

**DOI:** 10.1186/s13323-014-0015-6

**Published:** 2014-11-30

**Authors:** Anahit Hovhannisyan, Zaruhi Khachatryan, Marc Haber, Peter Hrechdakian, Tatiana Karafet, Pierre Zalloua, Levon Yepiskoposyan

**Affiliations:** Laboratory of Ethnogenomics, Institute of Molecular Biology NAS RA, 7 Hasratyan Str., Yerevan, Armenia; The Wellcome Trust Sanger Institute, Wellcome Trust Genome Campus, Hinxton, United Kingdom; Armenian DNA Project at Family Tree DNA, Houston, Texas 77008 USA; ARL Division of Biotechnology, University of Arizona, Tucson, Arizona 85721 USA; School of Medicine, Lebanese American University, PO Box 13–5053, Chouran, Beirut 1102 2801 Lebanon; Harvard School of Public Health, Boston, MA 02215 USA

**Keywords:** Armenian Highland, Y chromosome, Neolithic migration

## Abstract

**Background:**

The peopling of Europe and the nature of the Neolithic agricultural migration as a primary issue in the modern human colonization of the globe is still widely debated. At present, much uncertainty is associated with the reconstruction of the routes of migration for the first farmers from the Near East. In this context, hospitable climatic conditions and the key geographic position of the Armenian Highland suggest that it may have served as a conduit for several waves of expansion of the first agriculturalists from the Near East to Europe and the North Caucasus.

**Results:**

Here, we assess Y-chromosomal distribution in six geographically distinct populations of Armenians that roughly represent the extent of historical Armenia. Using the general haplogroup structure and the specific lineages representing putative genetic markers of the Neolithic Revolution, haplogroups R1b1a2, J2, and G, we identify distinct patterns of genetic affinity between the populations of the Armenian Highland and the neighboring ones north and west from this area.

**Conclusions:**

Based on the results obtained, we suggest a new insight on the different routes and waves of Neolithic expansion of the first farmers through the Armenian Highland. We detected at least two principle migratory directions: (1) westward alongside the coastline of the Mediterranean Sea and (2) northward to the North Caucasus.

**Electronic supplementary material:**

The online version of this article (doi:10.1186/s13323-014-0015-6) contains supplementary material, which is available to authorized users.

## Background

The large-scale transition from hunter-gathering to farming, known as the Neolithic Revolution, is broadly recognized as one of the crucial demographic events in human prehistory. It is considered that the advent of the *Neolithic lifestyle,* which is characterized by the dominance of settlement sedentism and the domestication of wild animals and plants, led to obvious advantages of farmers over hunter-gatherers and, in particular, drove dramatic human population growth and dispersal [1-3].

Archaeological research has uncovered the independent emergence of agricultural homelands in many parts of the world at different subsequent times, initially ranging between approximately 10 and 5 KYA [2,4]. In terms of chronology, the Fertile Crescent, the region in the Middle East, spanning the Zagros Mountains of Iran and Southern Mesopotamia northward to Southeast Anatolia, is widely recognized as the earliest farming center where agriculture is known to have originated, dating to around 10 KYA [5,6]. From the Fertile Crescent, human populations, with their cultural resources and languages, migrated towards various destinations, including Europe, currently the most thoroughly investigated region by archaeologists and geneticists [3,7].

Since the advance of molecular techniques, genetic studies have been extensively applied to disentangle a long-standing question about the nature of the spread of agriculture from the Fertile Crescent [8-11]. Under the demic diffusion model [5,8,12], the extant genetic diversity of Europeans would have resulted mainly from the genetic pool of the Near Eastern Neolithic farmers, while conversely, the cultural diffusion model asserts that European lineages would have been expected to have descended from indigenous hunter-gatherers [13-15]. In general, genetic studies based on different nuclear, mitochondrial, and Y-chromosomal markers and ancient DNA analysis differ considerably in their evaluation of the contribution of Paleolithic hunter-gatherers and Neolithic farmers to the composition of the modern European gene pool [16,17]. Recent discoveries indicating a third population, the Northern Eurasians, contributing their genetic legacy to modern Europeans, has further added to the complexity of these models [18]. Overall, previous studies highlight the entanglement and complexity of such historical events as farming dispersal and, ultimately, the peopling of Europe. The intricacies of these migratory events with varying patterns of cultural and demographic diffusion in different regions require the development of relevant models reflecting the process of Neolithic dispersal throughout Eurasia [7].

Despite the fast-growing application of the whole genomic sequencing approach on the reconstruction of human population history, convenient polymorphic markers of the non-recombining portion of the Y chromosome (NRY) still remain an indispensable and relatively simple tool for the patrilineal study of complex historic migration events that influenced modern-day Europeans’ genetic diversity [19-21]. In particular, relatively stable (in evolutionary terms) single-nucleotide polymorphisms (SNPs) with Y-chromosomal haplogroup defining characteristics and more rapidly mutating short tandem repeats (STRs) on the NRY locus are used in population genetic surveys for the detection of diversity among and within the studied populations [20]. Furthermore, among the useful features of the Y chromosome is its high level of geographic stratification and diversification, providing more specific inferences concerning population movement [22,23]. In addition to the frequency of classical genetic markers, the distribution of Y-chromosomal haplogroups shows broad clines across Europe, which was characterized as one of the main features of the European genetic landscape and regarded as evidence for the demic diffusion model [5]. Moreover, previous studies of Y-chromosomal haplogroup distribution reveal that the majority of contemporary European lineages fall into the haplogroups E, G, I, N, and R [20,24,25]. Further, it has been suggested that some Y-chromosomal haplogroups serve as specific markers of the Neolithic migration involving the first farmers from the Fertile Crescent, namely, E1b1b1-M35, J2-M172, G-M201, and R1b1a2-M269 lineages [22,24-26]. In particular, haplogroup R1b1a2-M269 is the most common Y-chromosomal lineage in Europe, encountered in 110 million European men, and increases in frequency westward [27,28]. Lately, the question of whether its origins were in the Paleolithic or Neolithic periods has become the subject of intense debate. In this context, Busby et al. claim that the existing data and methods are not capable of unambiguously estimating the age of its origin and the directions of its migration [29]. However, in some recent works, the observed explicit frequency cline of the haplogroup R1b1a2-M269 from Anatolia to Western Europe and its associated haplotype diversity cline in the opposite direction suggest that the lineage may have spread towards Europe with the migration of Neolithic farmers from the Near East [24,28]. Conversely, Y-chromosomal haplogroups G-M201 and J2-M172 are widely distributed in populations of the Caucasus, Near/Middle East, and Southern Europe, with the highest frequency in the North Caucasus [30,31]. These studies, however, did not consider the populations from the eastern regions of modern Turkey and the South Caucasus, roughly corresponding to the boundaries of the Armenian Highland, which could have served as a potential corridor for various Neolithic migrations.

Located at the crossroads of Europe and the Middle East, the Armenian Highland was a conduit for major waves of prehistoric and historic migrations [32], as well as a cradle for various ancient civilizations [33]. The unique geographic location of the plateau has garnered a great deal of scientific interest as a potential link between eastern and western Eurasian populations. Moreover, the variable climatic diversity and proximity to the Fertile Crescent likely contributed to the post-Last Glacial Maximum (LGM) Neolithic resettlements of the Armenian plateau, particularly by the first farmers from the Near East [32,34,35]. Dozens of archaeological and archaeobotanical artifacts related to agriculture and animal husbandry were discovered from the region, being consistent with the critical role of the Armenian Highland in the Neolithic farming migration from the Near East to Europe and the North Caucasus [36-38]. Though the area within the plateau is currently being studied by archaeologists, there is no convincing data enabling a proper description of the generalized pattern of Neolithic migrations through this region. However, it is possible to bridge this gap by applying the genetic study of populations indigenous to this geographic area. Here, we intended to identify the possible directions and waves of Neolithic migrations that had taken place via the Armenian Highland. To test the role of the region in the spread of Neolithic farmers, we studied the spatial frequency and diversity distribution of Y-chromosomal markers (drawing special attention to those linked with the spread of agriculturalists) in six geographically distinct Armenian populations, roughly covering the whole expanse of the Armenian Highland. Recently published genome-wide study results showing the absence of any significant admixture for Armenians over the past 4 KYA [39] justify using this population as a reference group for addressing the issue of Neolithic migration from the Near East to Europe and the North Caucasus.

## Methods

### Samples

**Figure 1:**
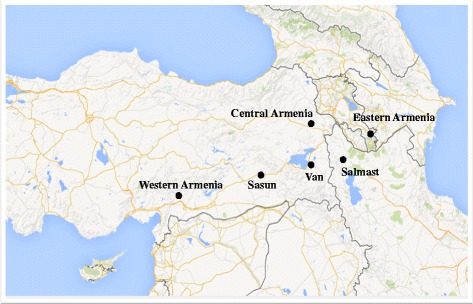
**Geographic locations of the Armenian populations studied.**

Buccal swabs were collected with informed consent from a total of 757 unrelated (at the paternal grandfather level) self-identified ethnic Armenian males, representing four geographically distinct Armenian regions of the historical expanse of Armenia. These regions include Salmast (*n* = 199), eastern (Karabakh and Syunik) (*n* = 210), central (Alashkert and Bayazet) (*n* = 200), and western (*n* = 148) parts of the Armenian plateau. All subjects were informed about the aim of this study and gave their consent to participate. The study protocol was approved by the Ethics Committee of the Institute of Molecular Biology NAS RA (IORG number 0003427, Assurance number FWA00015042, and IRB number 00004079). Further, in order to roughly encompass the whole region for analysis, we used previously published data for Van (*n* = 103), Sasun (*n* = 104), the Ararat Valley (*n* = 110), and Gardman (*n* = 96) [35], with the latter two, along with Karabakh and Syunik, then included in one group representing the eastern part of the Armenian Highland (Figure 1). To assess the frequency and diversity distribution of encountered Y-chromosomal haplogroups, we combined our data with previously published comparative datasets representing the Near East, the North Caucasus, and Europe. Overall, the present study comprises data from 35 populations (see Additional file 1).

### Y-SNP and Y-STR genotyping

**Figure 2:**
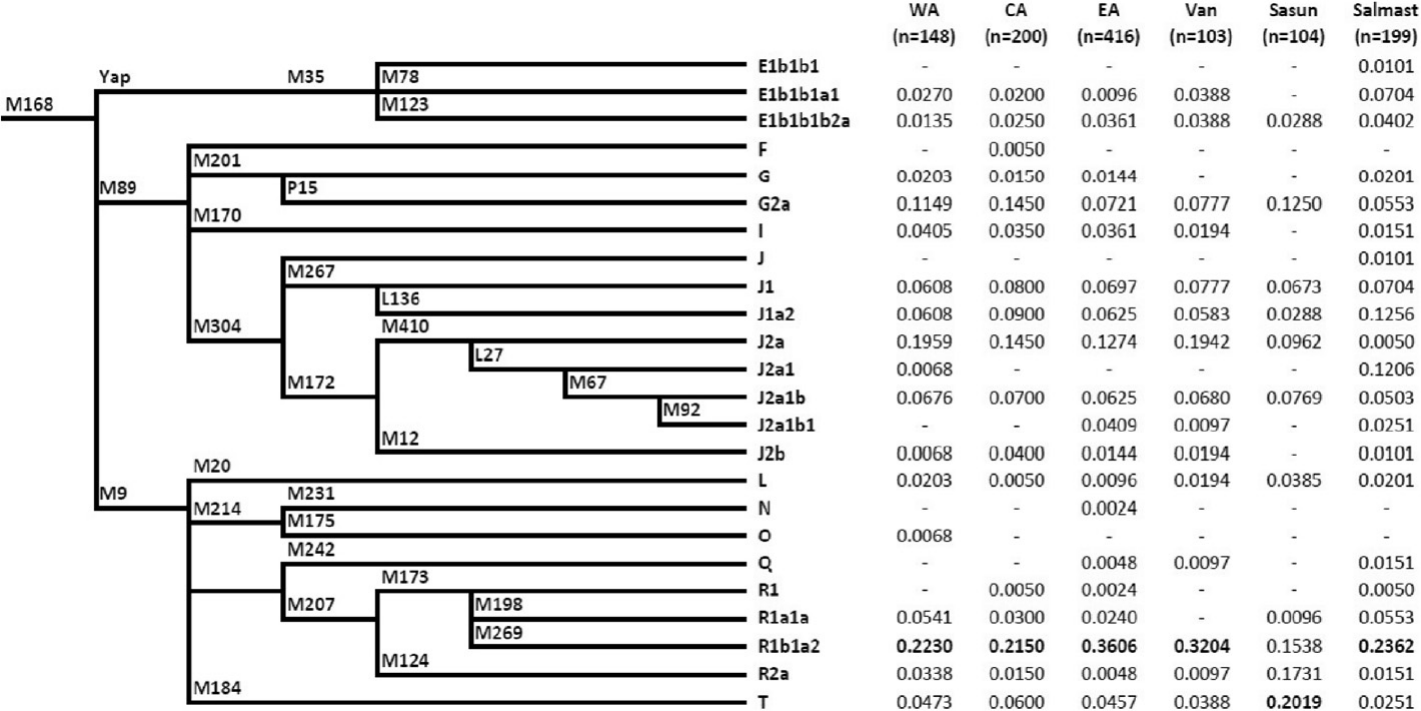
**Phylogenetic relationships and Y-chromosome haplogroup frequencies in six Armenian populations.**

The genotyping was performed in a hierarchical manner for the Y-chromosomal binary (SNP) markers and for STRs (see Additional file 2). The samples of western and central Armenia, Karabakh, and Syunik were genotyped at the Lebanese American University for 32 SNPs and 17 STRs. The genotyping of Salmast specimens was performed at the University of Arizona for 44 SNPs and 14 STRs. Nomenclature of Y-chromosomal haplogroups was assigned in accordance with ISOGG 2014 (http://www.isogg.org). In order to unify the number of haplogroups and STR markers while doing comparative analysis, we used 24 haplogroups for analysis within the Armenian populations (Figure 2), nine haplogroups**—**in comparison with other ethnic groups (see Additional file 3) and the following eight common STRs for all other cross-comparisons: DYS19, DYS389I, DYS389b, DYS390, DYS391, DYS392, DYS393, and DYS439.

### Data analysis

Measures of pairwise genetic distances (*F*_ST_) were calculated using the software package Arlequin 3.5 [40]. We also estimated the intra-population locus-specific variance, *V*_L_, and the intra-population genetic variance, *V*_P_, according to the formulae given in Kayser et al. [41]. Frequencies and microsatellite variances of the haplogroups were displayed using Surfer 10 (Golden Software) by the gridding method. Latitude and longitude values were calculated for the geographic centers of the sampling regions. Principal coordinate analysis (PCoA) was performed on distance matrices based on *F*_ST_ genetic distances using Genstat software. The phylogenetic relationships among eight loci haplotypes of equal number of individuals from different populations within the haplogroups R1b1a2, J2, and G were ascertained using the NETWORK 4.6.1.0 (available at http://www.fluxus-engineering.com) and Network Publisher softwares. Median-joining networks were generated by processing haplotypes with the reduced-median algorithm, followed by the median-joining method, and with weighted STR loci tabulated to be proportional to the inverse of the repeat variance. GENE-E software was used to graphically represent genetic similarities between populations by color coding pairwise *F*_ST_ values on a heatmap. To estimate differences in the haplogroup composition of the regions, correspondence analysis was conducted using SPSS ver. 19 software package (SPSS Inc.).

## Results and discussion

### Y-haplogroup frequency distribution

The phylogenetic relationships of Y-chromosomal markers and frequency distribution of the defined 24 haplogroups in the six Armenian populations are shown in Figure 2. The haplogroup R1b1a2-M269 is the most frequently encountered subclade in all Armenian samples, except Sasun, which differs from others due to the predominance of haplogroup T (20%) [35]. Of the lineages within haplogroup R, its subclade R1a1a-M198 is linked to the spread of Indo-Aryan languages [42] and detected with low frequencies or even absent in the analyzed populations. The majority of the J-M304 samples belongs to its J2-M172 branch, though in the population of Salmast, there is a nearly equal frequency distribution of J2 and J1 lineages. The haplogroup G is also observed at relatively high frequencies in all Armenian samples (Figure 2). On the whole, the results of analysis of patrilineal lineages revealed a prevalence of the Y-chromosomal haplogroups associated with the arrival of Neolithic farmers from the Near East. Three prospective genetic markers of agricultural migration, namely, the haplogroups J2, G, and R-M269, represent the most common lineages in all six Armenian populations, together accounting for 49%–70% of the sampled groups. It has previously been proposed that the wide presence of genetic markers attributed to agriculturalists, coupled with Neolithic archaeological artifacts, indicates continuous habitation of the Armenian Highland since the dawn of the Neolithic [32,35].

**Figure 3:**
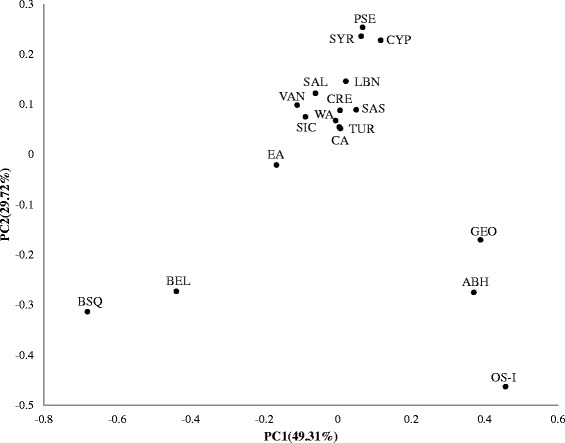
**PCoA plot based on pairwise** ***F***_**ST**_ **genetic distances calculated from haplogroup frequencies in the populations of this study.** The plot is based on *F*_ST_ pairwise genetic distances calculated from frequencies of nine common Y-chromosomal haplogroups (E1b1b1-M35, E(xE1b1b1), G-M201, I-M170, J2-M172, J(xJ2), L-M20, R1b1a2-M269, R(xR1b1a2)).

To obtain insight towards the question of the directions of movement for agriculturalists from the Near East, we used the PCoA method to visualize the *F*_ST_ genetic distances (based on absolute haplogroup frequencies in Additional file 3) between the Armenian and comparative datasets from the Near East, the North Caucasus, and Europe (see Additional file 4, sheet 1). The PCoA plot shows strong regional clustering indicating the separation of the populations from the Near East and Eastern Europe from those of the North Caucasus and Western Europe (Figure 3). In this context, populations of the Armenian Highland, the Near East, and Eastern Europe appear to be in one extensive cluster with a clear geographic gradient from the Levant towards the northwest. In fact, the closest population to the Near Eastern groups is Cyprus, the region settled by Neolithic farmers from the mainland shortly after the emergence of agriculture [43]. Moreover, the population of Crete hosts one of the oldest Neolithic settlements of Europe and underwent an agricultural transition from either the Anatolian coast or by sea from the Levant approximately 7–8 KYA [3,44]. The Cretan population within the cluster is centrally located between the populations of the Near East and Europe. This pattern is in accordance with previously found genetic affinity between human remains from Neolithic sites (based on aDNA data) and the modern populations of Cyprus and Crete, suggesting the leading role of pioneer seafaring colonization in the expansion towards the rest of Europe [17,45]. Specifically, our results of the PCoA analysis support a key role for Crete in the spread of Neolithic farmers through maritime routes from the Near East to Europe, which is also confirmed by pairwise *F*_ST_ value comparisons based on haplogroup frequencies (see Additional file 4, sheet 1). The plot on Figure 3 clearly separates the western European and North Caucasus populations from each other and bidirectionally from the Armenian cluster. These overall results further bolster the Armenian Highland as a corridor between the two aforementioned regions and the Near East.

**Figure 4:**
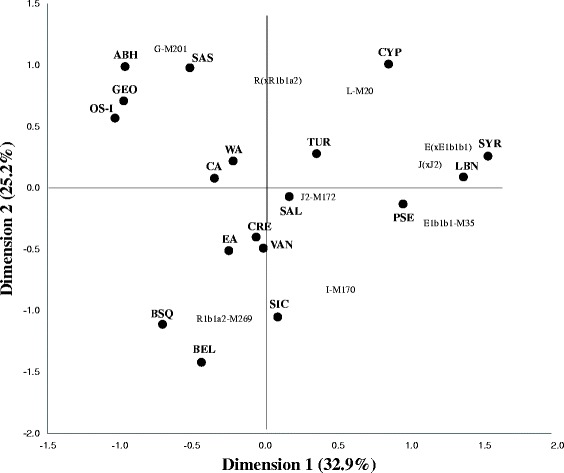
**Correspondence analysis plot based on the haplogroup frequency data in the populations studied.**

In order to provide a potential genetic explanation for the classification presented in Figure 3, we have conducted a correspondence analysis (Figure 4) on the haplogroup frequency data in the populations studied (Additional file 3). On the whole, the patterns of population distribution for correspondence analysis and PCoA are nearly identical. The European cluster, containing Basques, Sicilians, and Belgians, is associated with the haplogroups R1b1a2-M269 and I-M170, both widely spread in Europe, and the former being a marker for the Neolithic migration. The Caucasus cluster, comprising Abkhazians, Georgians, and Ossetians, is found to be connected to the haplogroup G-M201, which is also a marker for the Neolithic migration. The presence of the outlying Armenian population of Sasun in the vicinity of the Caucasus cluster could be explained by the geographic peculiarities of this high-mountainous group which lead to the genetic isolation from other Armenians during the intervening centuries. Completing the analysis of the haplogroups associated with the Neolithic agriculturalists, the lineage J2-M172 appears in between the European and Caucasus clusters.

The results of the PCoA and correspondence analysis show that the haplogroup composition of the Near Eastern populations is very similar to that found for the populations from the Anatolian and Armenian plateaus, as well as those of the Mediterranean islands. This is highly suggestive of a lengthy genetic continuity, persistent since at least the Neolithic. Apparently, the population migration of the first farmers from the Levant could have been both by land to Anatolia and the North Caucasus, and by maritime routes via eastern Mediterranean islands towards continental Europe. This scenario is supported by the result of the comparison of *F*_ST_ genetic distance values based on the frequencies of all haplogroups identified (see Additional file 4), showing that the populations of the Armenian Highland display an intermediate position between the Near East and Europe, and the Near East and the North Caucasus. Though previous work based on 15 autosomal STR loci from four Armenian populations (Ararat Valley, Gardman, Sasun, and Van) [46] derived a potential Balkan origin for one of these locations (Van), the results of our analysis not only support the transition zone model of the Armenian Highlands but also the potential gene flow of some Neolithic markers, shared among Armenians and Balkan populations, from the Near East through this region.

Further, in order to obtain deeper insight into the relationships between the populations observed and to analyze possible routes of expansion, we separately assessed the distribution patterns of putative Y-chromosomal tracers of the spread of the first agriculturalists. The values of frequency and genetic variance within each haplogroup among considered populations are provided in Additional file 5. Pairwise *F*_ST_ genetic distances and their statistical significances between the considered populations based on STR distribution within the haplogroups R1b1a2, J2, and G are available in Additional file 4 (sheets 2–4).

### Haplogroup R1b1a2-M269

The spatial distribution of the main western European Y-chromosomal lineage, haplogroup R1b1a2-M269, shows a significant frequency cline from 7% in Lebanon to 82% in Ireland [24,47], though also present in trace amounts in the majority of the North Caucasus populations [30]. Among Armenian samples, the haplogroup is one of the most common lineages, which is frequently encountered in the eastern part of the Armenian Highland and Van (see Additional file 5).

**Figure 5:**
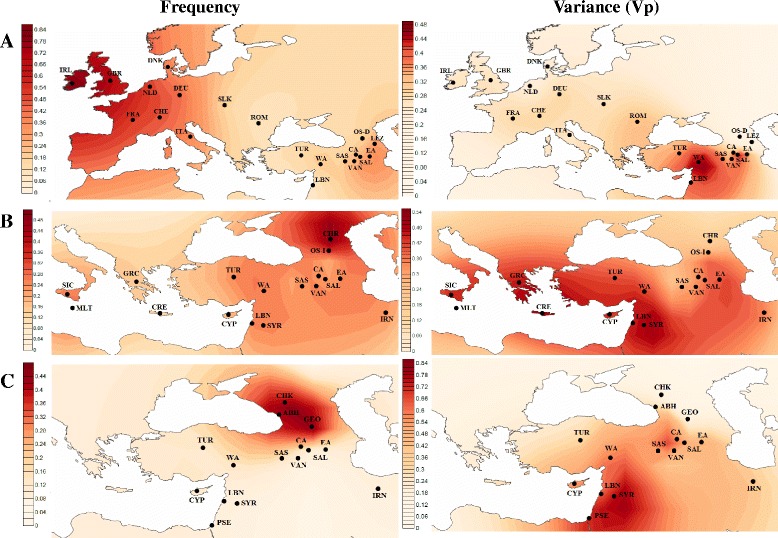
**Geographical distribution maps of haplogroup frequencies and genetic variances (** ***V***_**P**_ **): (A) R1b1a2, (B) J2, and (C) G.**

In contrast, a decreasing cline of microsatellite variance is detected from the Levant towards northwest and northeast. Furthermore, in comparison with all analyzed populations from the Near East, Europe, and Anatolia, the haplogroup R1b1a2-M269 occurs with the highest genetic variances in the western parts of the Armenian plateau, in Sasun and Salmast (Figure 5).

**Figure 6:**
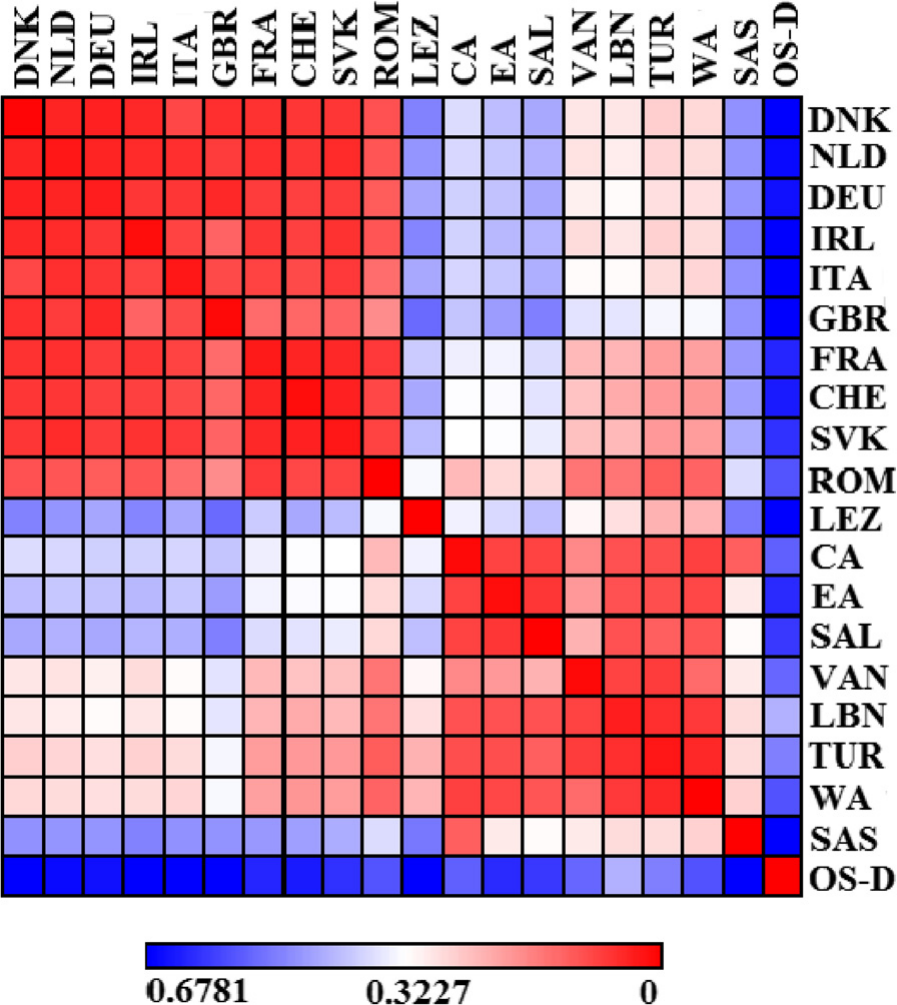
**Heatmap of pairwise** ***F***_**ST**_ **genetic distances, ranging from low (red) to high (blue), calculated for the haplogroup R1b1a2.**

A heatmap plot of *F*_ST_ distances within haplogroup R1b1a2 (Figure 6) reveals two large clusters with low genetic distances. The first represents a genetic homogeneity of European populations, while the second encompasses all populations of the Near East. Generally, only the population of Sasun is slightly different within the last group, likely due to the long centuries of its aforementioned isolation by geographic barriers. Moreover, in contrast to other populations of the Near Eastern cluster, the populations of the western part of the Armenian Highland, Van, Turkey, and Lebanon show a moderate level of genetic affinity to the central European populations. Indeed, the actual estimates of the *F*_ST_ values for haplogroup R1b1a2 place the western region of the Armenian Highland in a transitional position between the Near East and Europe (see Additional file 4, sheet 2). Previous data on the limited Y-chromosomal and autosomal sharing among the Armenian and European populations [31,35] should be considered as a consequence of the absence, in their Armenian datasets, of the populations from the western region of the Armenian Highland.

To assess the relationship between the haplotypes, we have conducted a median-joining network analysis within the haplogroup R1b1a2-M269 for the populations of Lebanon, the western part of the Armenian Highland, Italy, and Ireland, roughly approximating the path of human Neolithic migrations (see Additional file 6). The haplotypes of western Armenian origin are widely scattered and mainly associated with haplotypes from the Near Eastern (Lebanese) population. In addition, there are four haplotypes shared between Armenians and Europeans (Ireland and Italy), which was not revealed in Herrera et al. [35].

### Haplogroup J2-M172

The spatial distribution of haplogroup J2-M172 indicates highest encountered frequencies (>15%) in the areas between the Near East and the Mediterranean littoral [25,48]. Conversely, this lineage is also one of the most common haplogroups in the Caucasus (Figure 5) [30,49]. In particular, the lineage comprises 59% of the Y chromosomes in Chechen population and occurs with the lowest STR variance (14%), likely representing a strong founder effect signal [30]. Moreover, the distribution pattern of the haplogroup is consistent with a Levantine/Anatolian dispersal route to southeastern Europe and the Caucasus [25]. By this definition, the notion of ‘Anatolia,’ taken from Cinnioğlu et al. [50], actually includes the western and central areas of the Armenian Highland.

The frequency analysis of the haplogroup J2-M172 data within the Armenian populations shows that it is the most commonly encountered clade in the western and central parts of historical Armenia (27.7% and 25.5%, respectively)**.** Further, the western and eastern parts of the Armenian Highland have relatively high values of genetic variances, while the highest level among all populations was detected in Syria, in accordance with the suggested Near Eastern origin of this haplogroup (see Additional file 5) [25].

The heatmap plot of the *F*_ST_ values (see Additional file 7) within this haplogroup separates a distinct cluster of western Asian populations (Armenians, Turks, Lebanese, and Iranians). It also demonstrates a moderate level of genetic similarity between the majority of Armenian geographic groups (except Sasun) and the European populations. Our findings also indicate that western Armenians rather than eastern Armenians have a slightly closer genetic affinity with Greeks and Cretans based on the absolute values of pairwise *F*_ST_ distances. This result contradicts Herrera et al. [35], who demonstrated a segregation of Armenian populations from the European populations mentioned. In addition, eastern Armenians rather than western Armenians display closer genetic proximity to Ossets (relying on the *F*_ST_ values). On the whole, the comparison of *F*_ST_ genetic distances for haplogroup J2 indicates that the western Armenian population occupies an intermediate position between the Near East and Balkans on one hand, and Southern Europe on the other, while eastern Armenia serves as a genetic bridge between the Levant and the North Caucasus (see Additional file 4, sheet 3). Median-joining network analysis within the haplogroup J2 for the populations of Syria, western and eastern parts of the Armenian Highland, Crete, and Chechens also reflects the bidirectional split of the haplogroup J2 from the Near East westward and northward, mainly connecting western Armenia to Europe and eastern Armenia to the North Caucasus (see Additional file 8).

### Haplogroup G-M201

The Y-chromosomal haplogroup G-M201 is widely distributed in the populations of the Caucasus, the Near East, and Southern Europe, with the highest frequencies occurring in the North Caucasus (Figure 5) [30,31]. Our observations indicate that in the central part of the Armenian Highland, the haplogroup occurs with a relatively high frequency (16%), being inferior by this rate only to the populations of the North Caucasus. At the same time, the Armenian sample from the central region of the Armenian Highland has a comparable value of haplotype diversity (74.5%) with that of the Near Eastern populations of Syria (88.6%) and Palestine (79.3%) (see Additional file 5). Thus, our results support the recently published data on the origin of this haplogroup in the neighboring areas of eastern Anatolia, Armenia, and Western Iran [51].

The heatmap plot of *F*_ST_ values for the haplogroup G (see Additional file 9) does not identify distinct clusters of western Asian or European populations. Though the comparison of *F*_ST_ values does not conclusively indicate the intermediate position of the central part of the Armenian Highland for the Neolithic migration from the Near East to the North Caucasus, it does not reject this possibility either (see Additional file 4, sheet 4).

**Figure 7:**
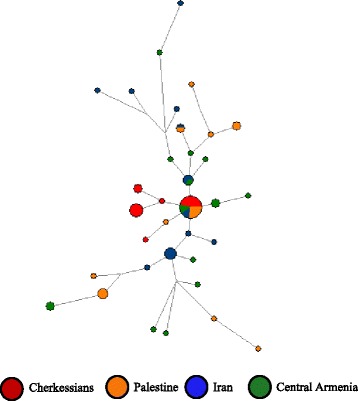
**Median-joining network of microsatellite haplotypes within the haplogroup G.** Circles represent microsatellite haplotypes, the areas of the circles are proportional to haplotype frequency (smallest circle corresponds to one individual), and population is indicated by color.

The constructed median-joining network within the haplogroup G (Figure 7) reveals the highest level of scattering of central Armenian haplotypes as compared to various neighboring populations (Palestinians, Cherkessians, Iranians), which is expected under the assumption of the local origin of this lineage. Furthermore, the network clearly shows the presence of a founder effect among the Cherkessian population of the North Caucasus who share their ancestral haplogroup with Armenians.

## Conclusions

Our observation of the Y-chromosomal structure in geographically different Armenian populations suggests that the Armenian Highland served as a transitional corridor for at least two distinct pathways of migration for Neolithic farmers from the Near East westward and northward. The movement to Europe took place predominantly via the western region of the Armenian Highland alongside the coastline of the Mediterranean Sea, which is supported by the spatial distribution pattern of the haplogroup R1b1a2-M269. The migration to the North Caucasus occurred mainly across the central and eastern regions of the Armenian Highland, which is shown by the geographical distribution of haplogroup G-M201. In addition, we identified a distinct Neolithic wave of bidirectional expansion to Europe and the North Caucasus associated with haplogroup J2-M172.

**Figure 8:**
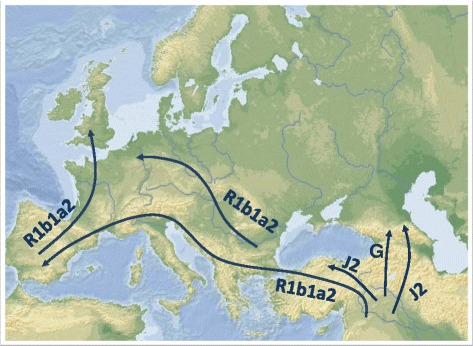
**Different waves and directions of Neolithic migration from the Fertile Crescent.**

Thus, at the initial stage of the Neolithic migration from the Levant, different directions and waves of population movement could be identified in the Armenian Highland (Figure 8). This inference needs to be tested by further study of other indigenous populations of the region using higher resolution genotyping of Y-chromosome, mitochondrial, and autosomal DNA markers, as well as applying the data recovered from ancient DNA.

## Additional files

Additional file 1:
**List of populations analyzed in this study.** Samples used as comparative datasets representing the Near East, the North Caucasus, and Europe. Additional file 2:
**Typing results of the Y-chromosomal SNP and STR markers in the Armenian samples studied.** List of haplogroup distribution and STRs for the haplogroups R1b1a2, J2, and G. Additional file 3:
**Absolute frequency distribution of haplogroups in the studied populations.** Frequencies of the main Y-chromosomal haplogroups in the 18 populations included in the PCoA and correspondence analysis of Figures 3 and 4. Additional file 4:
**Pairwise** ***F***_**ST**_ **genetic distances between the populations studied.** Pairwise *F*_ST_ genetic distances between the populations studied based on all haplogroup frequencies, and for the haplogroups R1b1a2, J2, and G. Additional file 5:
**Distribution of the haplogroups R1b1a2, J2, and G.** The values of frequency and genetic variance within the haplogroups R1b1a2, J2, and G among the considered populations. Additional file 6:
**Median-joining network of microsatellite haplotypes within the haplogroup R1b1a2.** Circles represent microsatellite haplotypes, the areas of the circles are proportional to haplotype frequency (smallest circle corresponds to one individual), and population is indicated by color. Additional file 7:
**Heatmap of pairwise** ***F***_**ST**_ **genetic distances between the studied populations calculated for the haplogroup J2.** Pairwise *F*_ST_ genetic distances on the heatmap range from low (red) to high (blue). Additional file 8:
**Median-joining network of microsatellite haplotypes within the haplogroup J2.** Circles represent microsatellite haplotypes, the areas of the circles are proportional to haplotype frequency (smallest circle corresponds to one individual), and population is indicated by color. Additional file 9:
**Heatmap of pairwise** ***F***_**ST**_ **genetic distances between the studied populations calculated for the haplogroup G.** Pairwise *F*_ST_ genetic distances on the heatmap range from low (red) to high (blue).

## Abbreviations

KYA: Kilo years ago
NRY: Non-recombining portion of the Y chromosome
SNP: Single-nucleotide polymorphism
STR: Short tandem repeat
LGM: Last glacial maximum
PCoA: Principal coordinate analysis
