# Supplementary figures and images for: Different waves and directions of Neolithic migrations in the Armenian Highland
Source: Investig Genet. 2014 Nov 30;5:15. doi: 10.1186/s13323-014-0015-6 (PMC4249771; doi:10.1186/s13323-014-0015-6)

## Slide 1
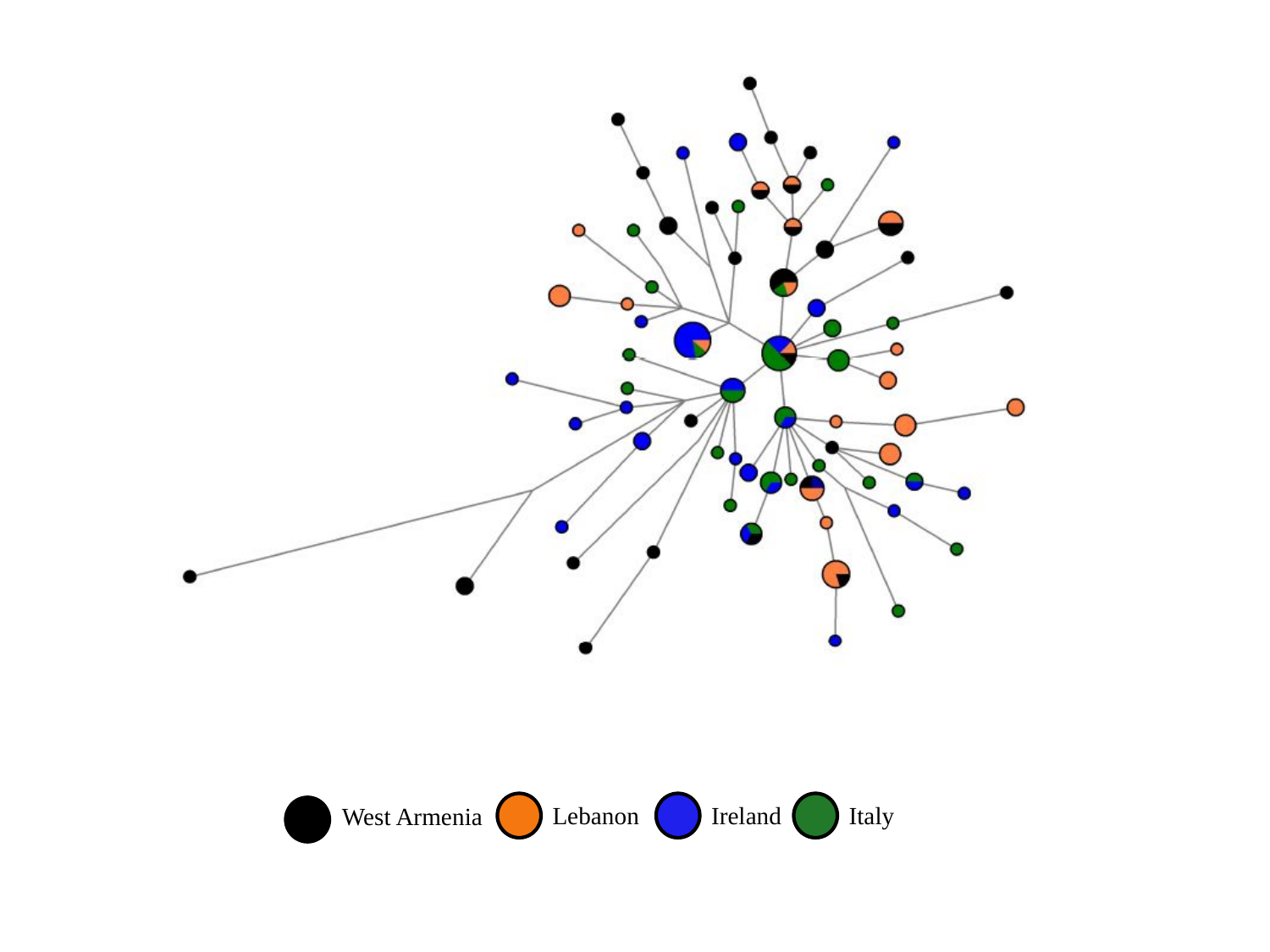

Lebanon
Ireland
Italy
West Armenia

Supplement: Additional file 6: — Median-joining network of microsatellite haplotypes within the haplogroup R1b1a2. Circles represent microsatellite haplotypes, the areas of the circles are proportional to haplotype frequency (smallest circle corresponds to one individual), and population is indicated by color. [file 13323_2014_15_MOESM6_ESM.pptx]

## Slide 1
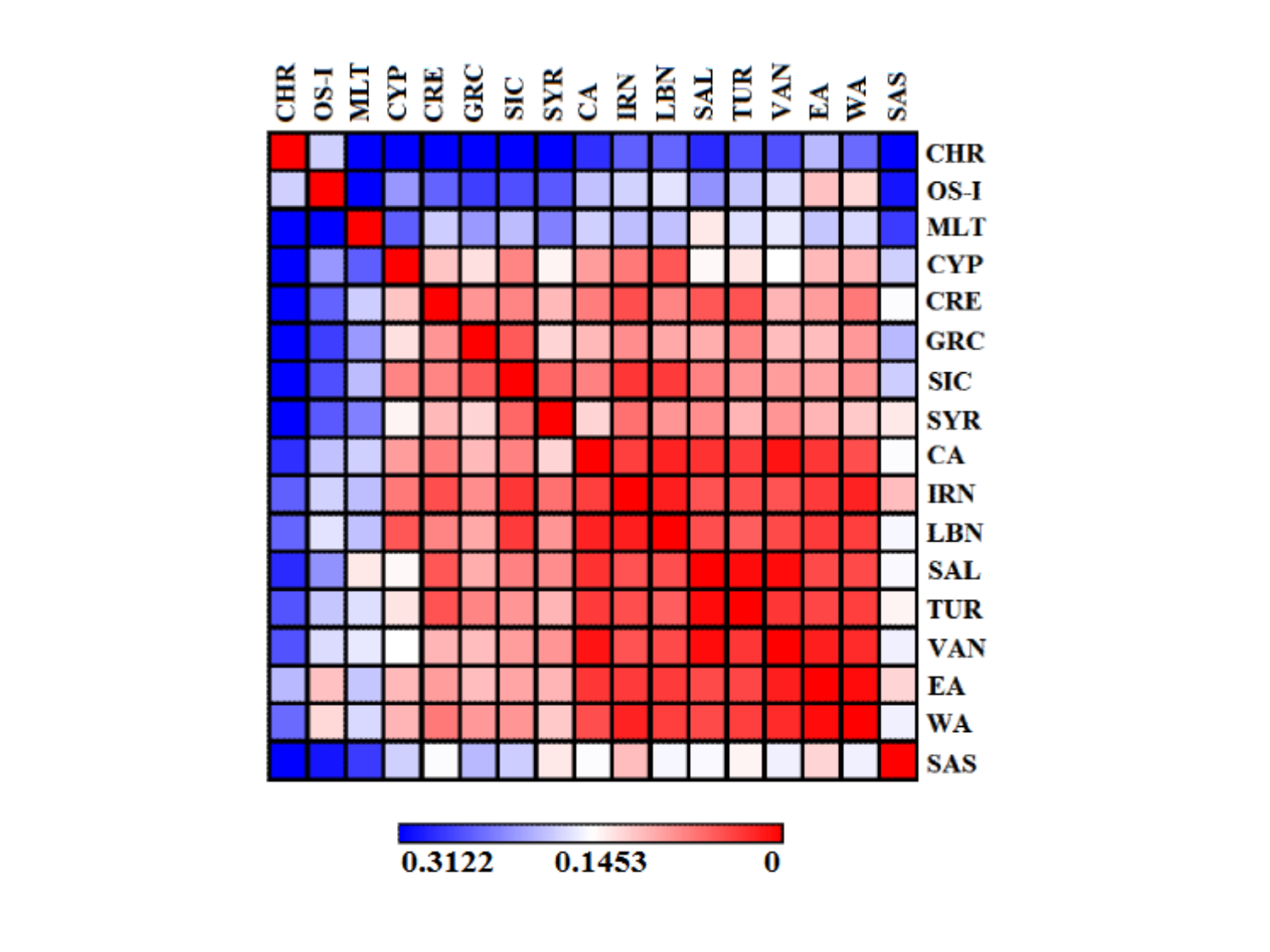

Supplement: Additional file 7: — Heatmap of pairwise F ST genetic distances between the studied populations calculated for the haplogroup J2. Pairwise F ST genetic distances on the heatmap range from low (red) to high (blue). [file 13323_2014_15_MOESM7_ESM.pptx]

## Slide 1
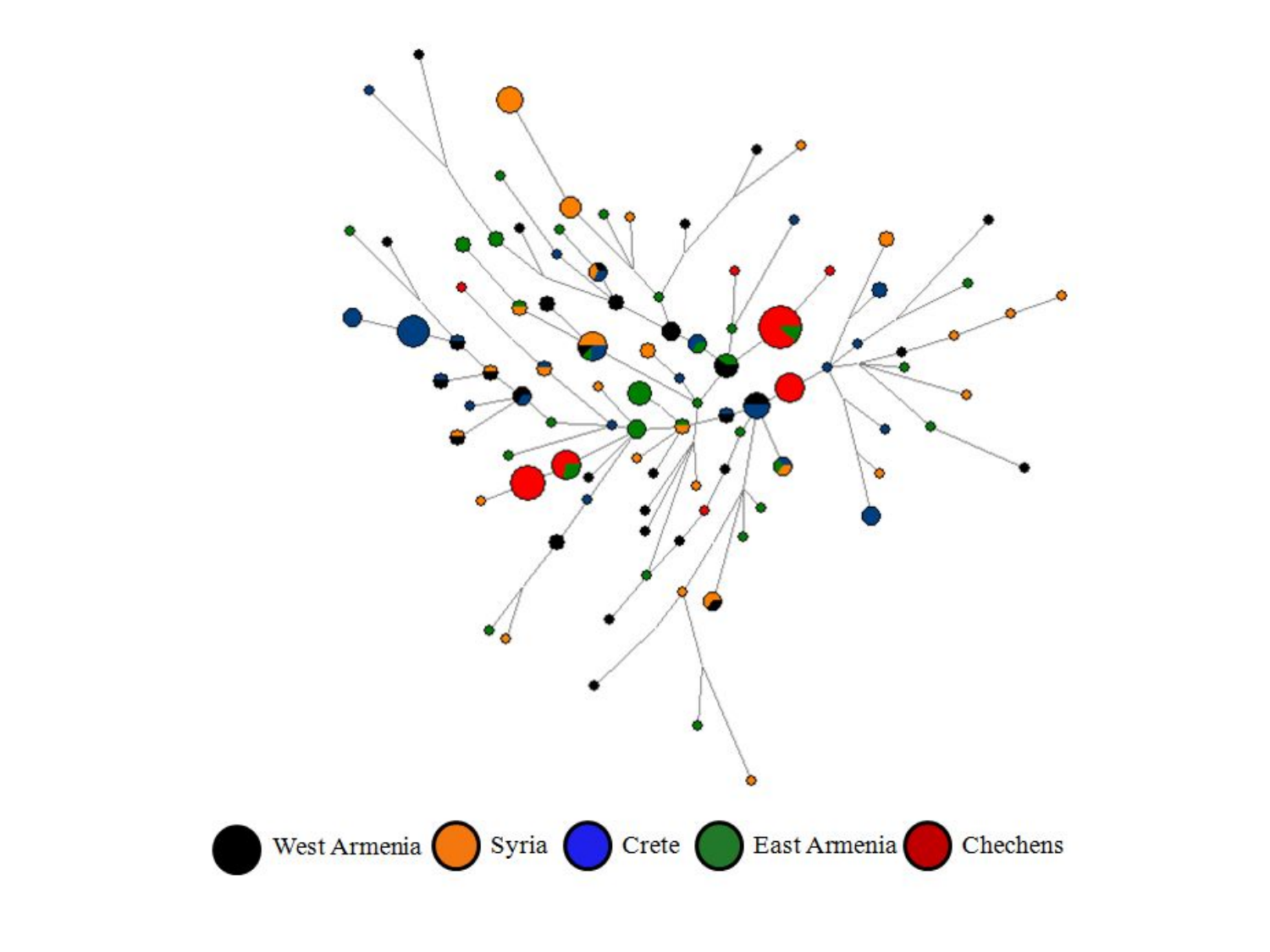

Supplement: Additional file 8: — Median-joining network of microsatellite haplotypes within the haplogroup J2. Circles represent microsatellite haplotypes, the areas of the circles are proportional to haplotype frequency (smallest circle corresponds to one individual), and population is indicated by color. [file 13323_2014_15_MOESM8_ESM.pptx]

## Slide 1
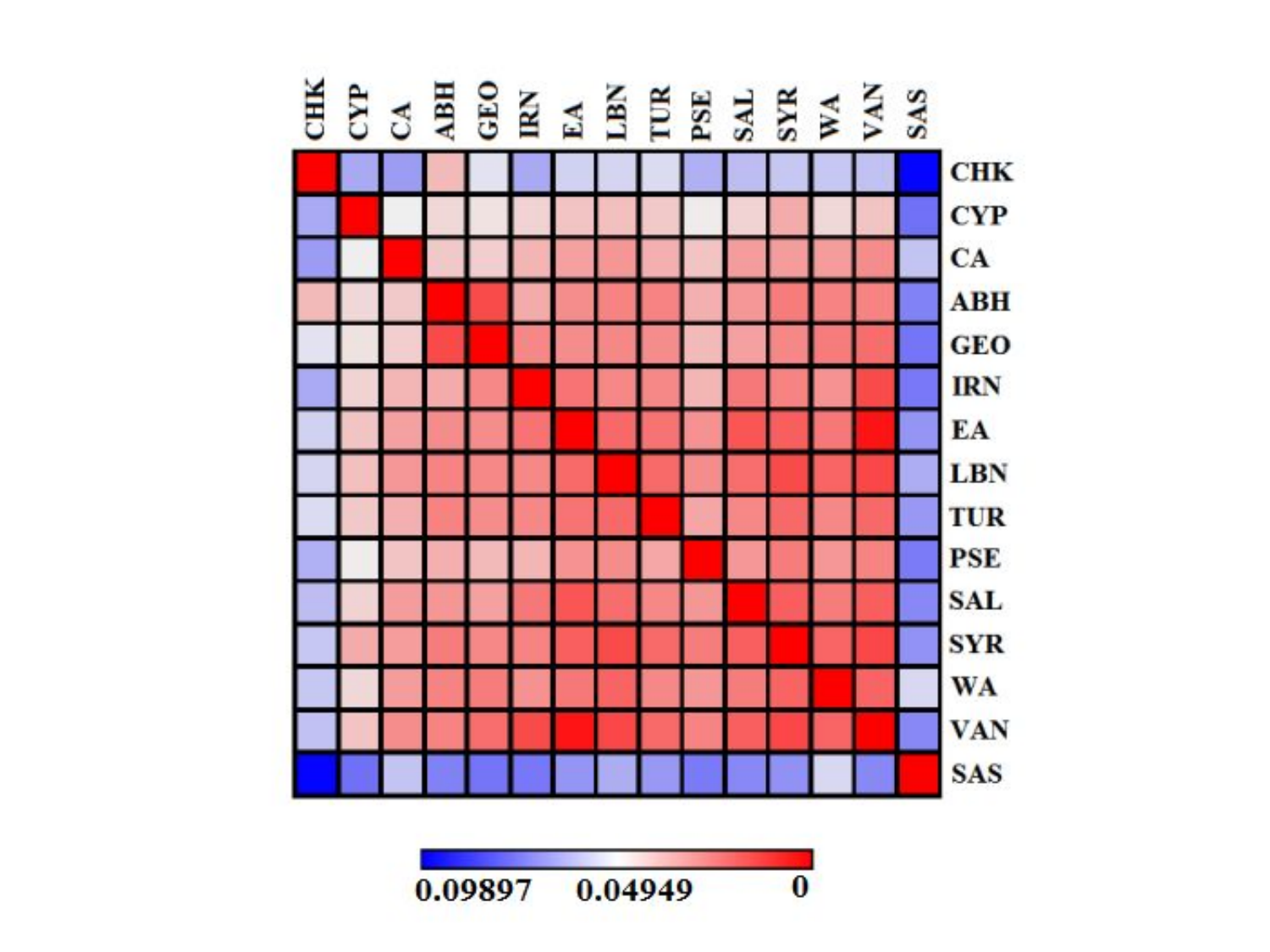

Supplement: Additional file 9: — Heatmap of pairwise F ST genetic distances between the studied populations calculated for the haplogroup G. Pairwise F ST genetic distances on the heatmap range from low (red) to high (blue). [file 13323_2014_15_MOESM9_ESM.pptx]
